# Antibacterial activity of green-synthesized silver nanoparticles against Gram-negative bacteria and insights into potential resistance mechanisms

**DOI:** 10.36922/itps025080007

**Published:** 2025-07-01

**Authors:** Akamu J. Ewunkem, Bliss Daodu, Zahirah J. Williams, Lydia Merrills, Brittany L. Justice, Felicia Simpson, David Holland, Tatyana Bowers, Uchenna Iloghalu

**Affiliations:** 1 Department of Biological Sciences, Faculty of Natural and Physical Sciences, Winston-Salem State University, Winston-Salem, North Carolina, United States of America; 2 Department of Nursing, Faculty of Natural and Physical Sciences, Winston-Salem State University, Winston-Salem, North Carolina, United States of America; 3 Department of Mathematics, Faculty of Natural and Physical Sciences, Winston-Salem State University, Winston-Salem, North Carolina, United States of America

**Keywords:** Carpenter bee wing extracts, Genomics, Gram-negative bacteria, Green synthesis, Nanoparticles

## Abstract

Gram-negative bacterial infections pose a serious public health challenge due to their high global mortality rates and potential to cause severe complications. Antibiotics – one of the most impactful medical innovations of the 20^th^ century – remain vital in treating life-threatening bacterial infections. However, the increasing prevalence of antibiotic resistance has made it progressively harder to treat Gram-negative bacterial infections effectively. Therefore, nanoparticles have gained attention as a promising alternative treatment owing to their targeted antibacterial properties. Among the various synthesis methods, green synthesis is considered one of the most effective approaches for nanoparticle production. In this study, silver nanoparticles were synthesized using a green approach that utilized silver nitrate salt and an extract derived from carpenter bee wings (CBWs). The synthesized nanoparticles were characterized using spectroscopic techniques and scanning electron microscopy. Their antibacterial activity was tested against two pathogenic Gram-negative bacteria using the broth dilution method. Furthermore, whole genome sequencing was conducted to assess the mutagenic effects of the biosynthesized silver nanoparticles on the two bacterial strains. The results demonstrated that the green-synthesized silver nanoparticles exhibit notable antibacterial activity, likely through electrostatic interactions that promote cell binding and induce significant morphological alterations. Genomic analysis revealed mutations associated with efflux pump regulation, neutralization, transport, energy metabolism, cell division, biosynthetic pathways, adaptation, and invasion in the tested strains. These findings demonstrate the potential of CBWs as a novel biological resource for the green synthesis of silver nanoparticles with antibacterial properties. However, the study also raises concerns regarding the potential for bacteria to develop resistance to nanoparticles over time.

## 1. Introduction

Gram-negative bacterial infections are among the leading causes of both hospital-acquired and community-acquired infections, commonly seen in diseases such as septicemia, tuberculosis, and pneumonia. Among the most prevalent Gram-negative pathogens are *Klebsiella pneumoniae* and *Escherichia coli*.^1^ These bacteria are responsible for a wide range of infections affecting various body systems, including the gastrointestinal tract, renal system, and central nervous system.^2–5^ Effective treatment of *K. pneumoniae* and *E. coli* infections is crucial, as delayed or inadequate treatment can result in severe complications and potentially fatal outcomes. Therefore, timely medical intervention is critical to manage infections caused by *K. pneumoniae* and *E. coli*.

Antibiotics represent one of the most impactful medical innovations in modern history. Antibiotics – such as chloramphenicol, glycylcyclines, fluoroquinolones, cephalosporins, and aminoglycosides – play a crucial role in the treatment of *K. pneumoniae* and *E. coli* infections, saving millions of lives worldwide.^6–8^ These drugs target specific processes or structures within bacterial cells, thereby disrupting essential cellular functions. Depending on their mechanism of action, antibiotics can exhibit bacteriostatic effects (inhibiting bacterial growth) or bactericidal effects (killing bacteria).^9,10^ However, the widespread and reckless use of antibiotics has significantly contributed to the emergence of antimicrobial resistance.^11^

Gram-negative bacteria – including *E. coli* and *K. pneumonia* – have developed multiple resistance mechanisms against antibiotics.^12–14^ One major defense is their outer membrane, which acts as a barrier to hinder antibiotic penetration.^13^ In addition, the production of β-lactamases confers resistance to a wide range of antibiotics, such as penicillin, cephalosporins, and carbapenems.^15^ Resistance is further enhanced by mutations or deletions in porin proteins such as OmpK35 and OmpK36, which reduce the uptake of antimicrobial agents.^16^ Bacterial resistance presents a major public health threat, leading to severe infections and is projected to contribute to approximately 10 million deaths annually by 2050.^17^ This highlights the urgent need to explore innovative alternatives to traditional antibiotic treatments.

Nanoparticles emerge as a promising alternative to antibiotics for treating bacterial infections, largely due to their ability to overcome microbial drug resistance.^18^ They exhibit antimicrobial activity by directly interacting with and disrupting bacterial cell membranes through mechanisms such as physical penetration and generating reactive oxygen species, ultimately leading to cell damage and death.^19^

Their small size enables close interaction with bacterial membranes, causing structural damage and leakage of cellular contents, ultimately leading to cell death.^20^ Furthermore, metal-based nanoparticles can penetrate bacterial cells and interact with intracellular components – including proteins, nucleic acids, and lipids – disrupting essential cellular processes. These interactions may also induce mutations and contribute to cell death due to their high surface area.^21^

The antimicrobial activity of silver nanoparticles has been extensively explored against a wide range of pathogenic bacteria, including *E. coli* and *K. pneumonia*.^22–26^ Previous research has also examined the antibacterial effects of silver nanoparticles synthesized using the extract from the wings of carpenter bees (*Xylocopa virginica*) – hereafter referred to as carpenter bee wings (CBWs) – against selected Gram-negative and Gram-positive bacteria.^24^ These biosynthesized silver nanoparticles have been shown to exert antibacterial effects by aggregating on bacterial cell surfaces, potentially interacting with cellular components in ways that lead to mutations. These findings raise important concerns about the long-term risk of bacterial adaptation or resistance in response to nanoparticle-based antimicrobial strategies.

However, the specific mutations associated with biologically synthesized silver nanoparticles in Gram-negative bacteria remain largely unexplored. This study hypothesizes that *X. virginica* wing extract can be used to synthesize silver nanoparticles with improved antimicrobial activity and the potential to induce genetic changes in target bacteria.

The findings from this study may provide valuable insights into the mechanisms by which biosynthesized nanoparticles exert antimicrobial effects and how they may contribute to the development of bacterial resistance. In particular, identifying mutations in genes related to cell wall integrity, DNA repair, and stress response pathways could help elucidate bacterial adaptation strategies. Understanding these mutations can guide the development of optimized nanoparticles with reduced potential to induce resistance, thereby enhancing their effectiveness in healthcare and environmental applications.

This study aims to evaluate the *in vitro* antimicrobial activity of green-synthesized silver nanoparticles produced using *X. virginica* wing extract against two pathogenic Gram-negative bacterial strains, namely *E. coli* and *K. pneumoniae*. The morphology and size distribution of the synthesized nanoparticles are characterized using spectroscopic analyses. In addition to assessing their antibacterial activity, the study investigates the potential of these biosynthesized silver nanoparticles to induce genetic mutations in the target bacteria – an effect that raises important concerns regarding the possible development of resistance to nanoparticle-based antimicrobial agents.

## 2. Materials and methods

### 2.1. Materials

In this study, carpenter bees were obtained from Winston-Salem State University. The two Gram-negative bacterial strains – *E. coli* 1946 and *K. pneumonia* – were obtained from the American Type Culture Collection (ATCC; United States). The following analytical-grade chemicals were used: deionized water, 70% ethanol (Fisher Scientific, USA), 1 mM silver nitrate (Fisher Scientific, USA), 0.1 M sodium hydroxide (Fisher Scientific, USA), nutrient broth (Fisher Scientific, USA), phosphate-buffered saline (Fisher Scientific, USA), and glutaraldehyde solution (Fisher Scientific, USA). Additional materials included a 98-well plate (Fisher Scientific, USA), centrifuge tubes (Fisher Scientific, USA), Ziploc bags, a field emission scanning electron microscope (JEOL Ltd., Japan), a GENESYS^™^ 180 ultraviolet-visible (UV-vis) spectrophotometer (Fisher Scientific, United States), a DNeasy 96 PowerSoil Pro QIAcube HT Kit (QIAGEN, USA), and an Isotemp 2300 Digital Water Bath (Fisher Scientific, USA).

### 2.2. Extract preparation and synthesis of silver nanoparticles

Dead carpenter bees (*X. virginica*) were collected in June 2022 from Winston-Salem State University, Winston-Salem, North Carolina, United States of America, and transported to the laboratory in Ziploc bags. The wings were carefully removed using sterilized forceps, then sterilized in 70% ethanol, washed with deionized water, and air-dried at room temperature.

Silver nanoparticles were synthesized using the following protocols adapted from previous studies.^24,25^ Briefly, 0.1 g of bee wings was weighed and hydrolyzed in 0.1 M sodium hydroxide at 90°C using an Isotemp 2300 Digital Water Bath for 60 min. After hydrolysis, the mixture was cooled and centrifuged at 8,000 rpm for 10 min. The supernatant pH was adjusted to neutral, and 1 mL of this extract was added to 49 mL of 1 mM silver nitrate solution in a 100 mL beaker. The mixture was incubated at 28 ± 1°C for 60 min, during which the color change from light yellow to dark brown indicated the formation of silver nanoparticles. The UV-vis absorbance spectrum of the synthesized nanoparticles was measured in the 200 – 1,000 nm range using a GENESYS^™^ 180 UV-vis spectrophotometer. Scanning electron microscopy (SEM) was performed using a field emission SEM to characterize the morphology and size distribution of the nanoparticles.

### 2.3. Antibacterial analysis

*E. coli* 1946 (ATCC 25922) and *K. pneumoniae* NCTC 9633 (ATCC 13883) were cultured in nutrient broth medium at 37°C for 24 h with shaking at 150 rpm in a shaking incubator. The antibacterial activity of biosynthesized silver nanoparticles from CBW at concentrations ranging from 0 to 100 μM was evaluated against *E. coli* and *K. pneumoniae*, following protocols adapted from previous studies.^24,25^ After 24 h of incubation, bacterial growth was assessed by measuring the optical density at 600 nm using a 98-well plate format with a GloMax Multiplate Reader (Promega, United States). In addition, SEM was used to analyze the morphological changes in the treated bacterial strains_._^24,25^

### 2.4. Genomic analysis

Whole genome sequencing (WGS) was performed to investigate the genetic alterations in *E. coli* and *K. pneumoniae* following exposure to biosynthesized silver nanoparticles.^25,26^ Briefly, after 24 h of treatment, genomic DNA was extracted from both the control group (untreated bacteria) and the nanoparticle-treated cells using the DNeasy 96 PowerSoil Pro QIAcube HT Kit, following the instructions provided by the manufacturer. Genomic libraries were prepared and sequenced using the NextSeq2000 system (manufacturer, country) with a 300-cycle flow cell kit to generate 2 × 150 base pair paired-end reads. Read demultiplexing, trimming, and run analytics were performed using DRAGEN v4.2.7, the onboard analysis software integrated with the NextSeq2000 system.

### 2.5. Statistical analysis

All statistical analyses were conducted using GraphPad Prism version 8.01 (manufacturer, country). Data are presented as the mean ± standard error of the mean. Statistical comparisons between groups were conducted using the Student’s *t*-test, and differences were considered statistically significant at *p*<0.05.

## 3. Results

### 3.1. Characterizations of biosynthesized silver nanoparticles from CBWs

**Figure 1.**
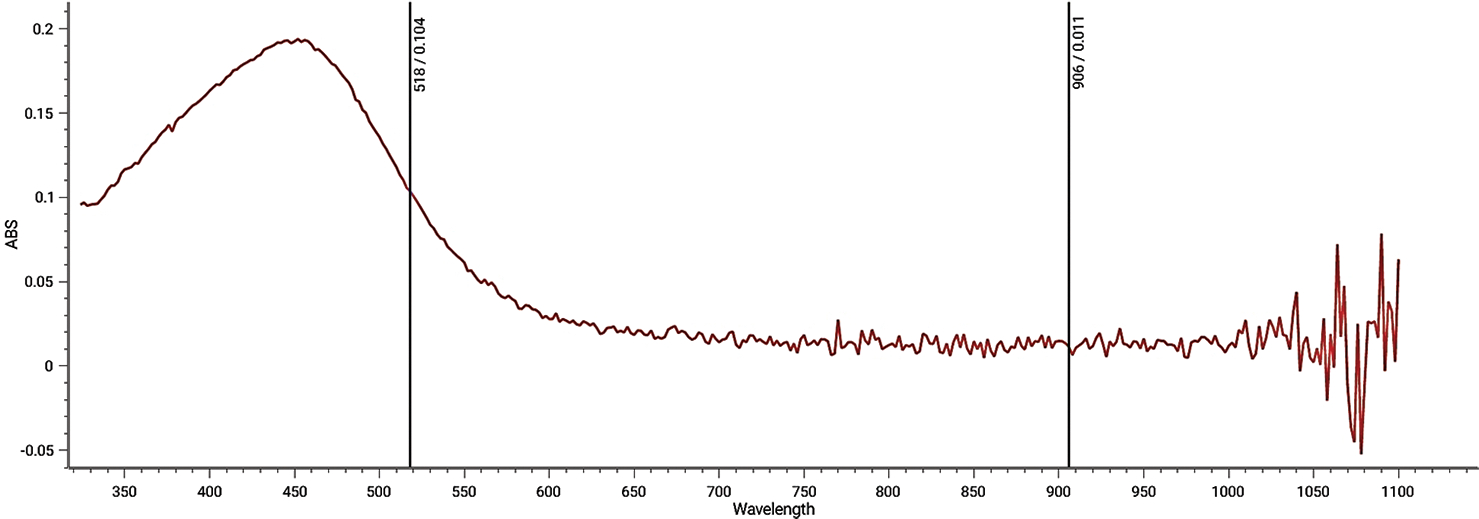
Ultraviolet-visible spectra of biosynthesized silver nanoparticles from carpenter bee wing extract

The biosynthesis of silver nanoparticles is visually confirmed by a color change in the treated extract, turning from light yellow to dark brown. The UV-vis spectral analysis reveals a strong surface plasmon resonance (SPR) peak at approximately 420 nm, indicating the successful formation of silver nanoparticles (Figure 1).^25^

**Figure 2.**
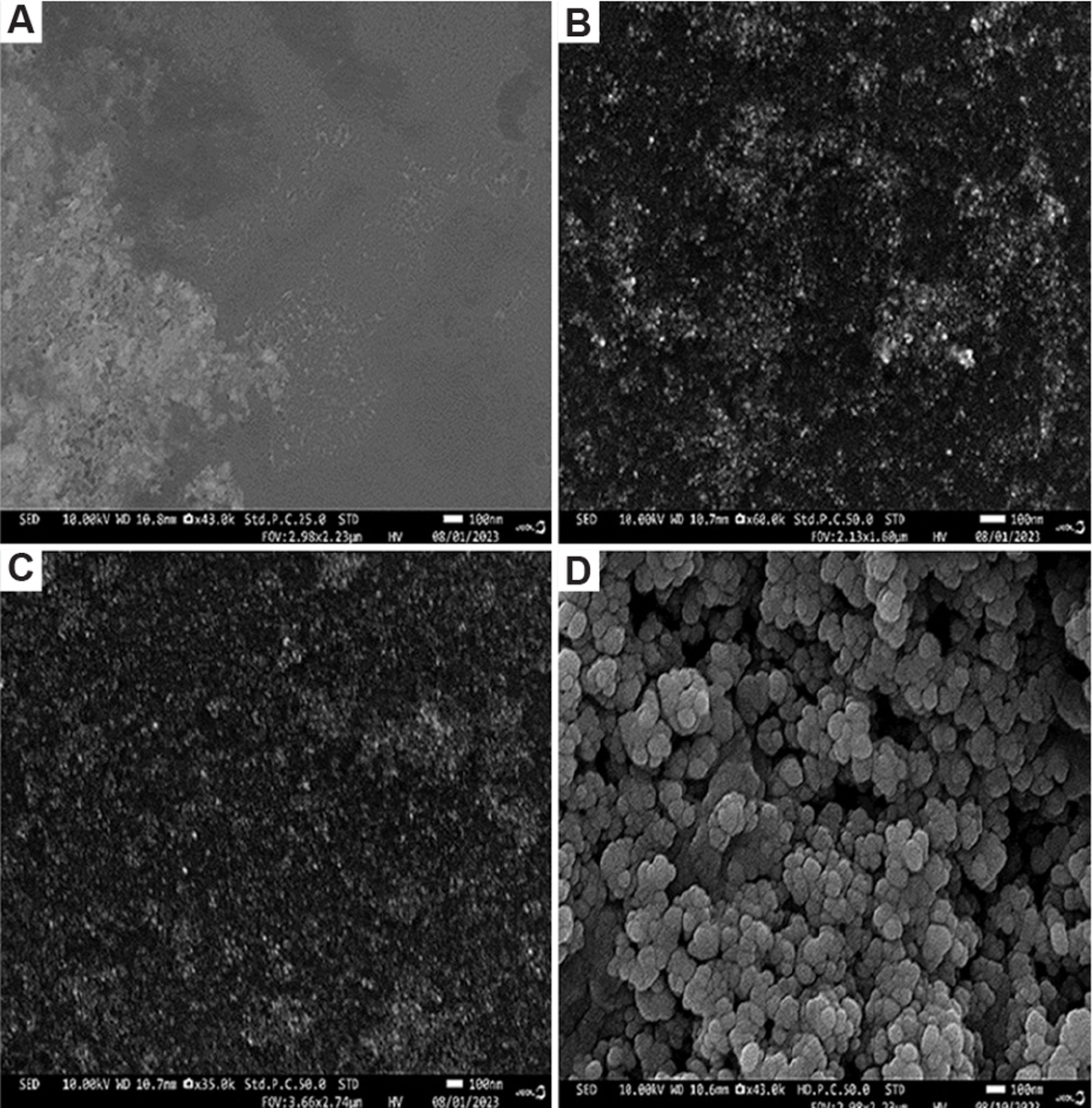
Scanning electron microscopy micrographs of biosynthesized silver nanoparticles from carpenter bee wing extract, each displaying a 100 nm scale bar and captured at different magnifications: (A) ×43,000, (B) ×60,000, (C) ×35,000, and (D) ×43, 000

The shape, size, morphology, and composition of the synthesized nanoparticles directly influence the SPR bands. SEM images reveal agglomeration of the biosynthesized silver nanoparticles (Figure 2). In addition, SEM analysis shows that the nanoparticles exhibit a spherical morphology with an approximate diameter ranging from 10.0 nm to 40.0 nm (Figure 2).

**Figure 3.**
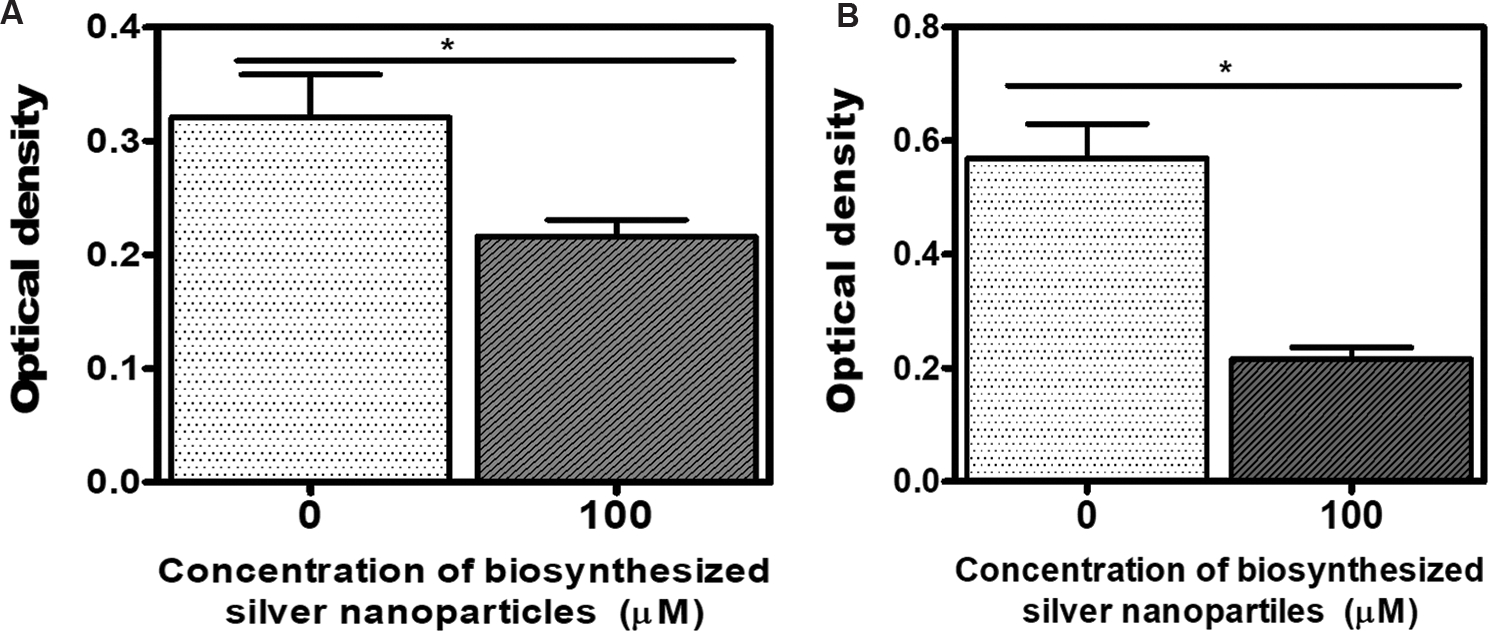
Antibacterial activity of biosynthesized silver nanoparticles from carpenter bee wing extract against (A) *Escherichia coli* and (B) *Klebsiella pneumoniae* after 24 h of exposure Note: Asterisk (*) indicates statistically significant differences compared to the control (*p*<0.05).

**Figure 4.**
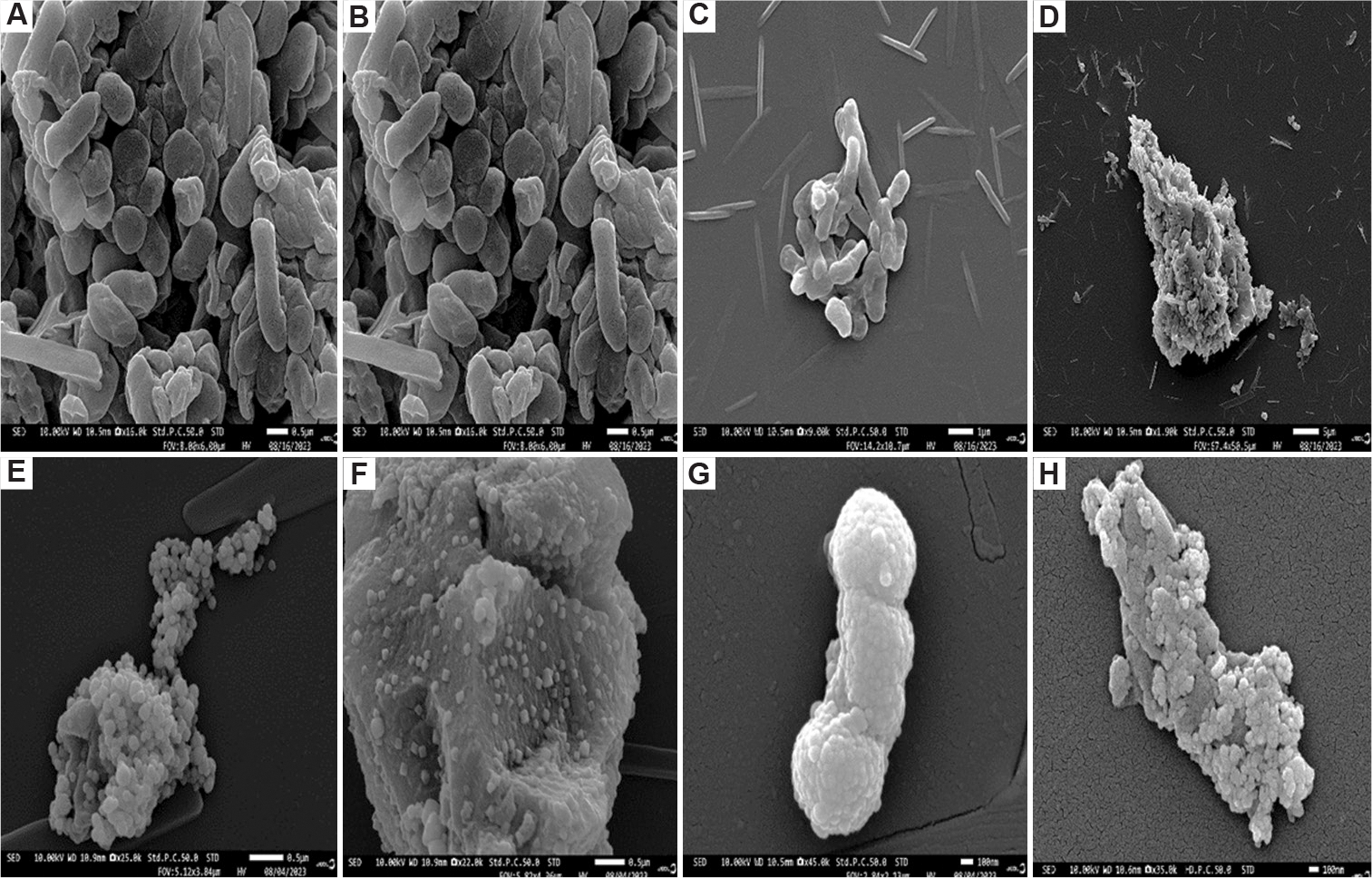
Scanning electron microscopy images of *Klebsiella pneumoniae* cells show interactions with biosynthesized silver nanoparticles from carpenter bee wing extract after 24 h of exposure. (A-D) Control (untreated) cells; (E-H) cells treated with the silver nanoparticles. (A and B) Scale bar = 0.5 μm, magnification = ×15,000; (C) scale bar = 1 μm, magnification = ×9,000; (D) scale bar = 5 μm, magnification = ×1,900; (E) scale bar = 0.5 μm, magnification = ×25,000; (F) scale bar = 0.5 μm, magnification = 22,000×; (G) scale bar = 100 nm, magnification = 45,000×; (H) scale bar = 100 nm, magnification = ×35,000.

**Figure 5.**
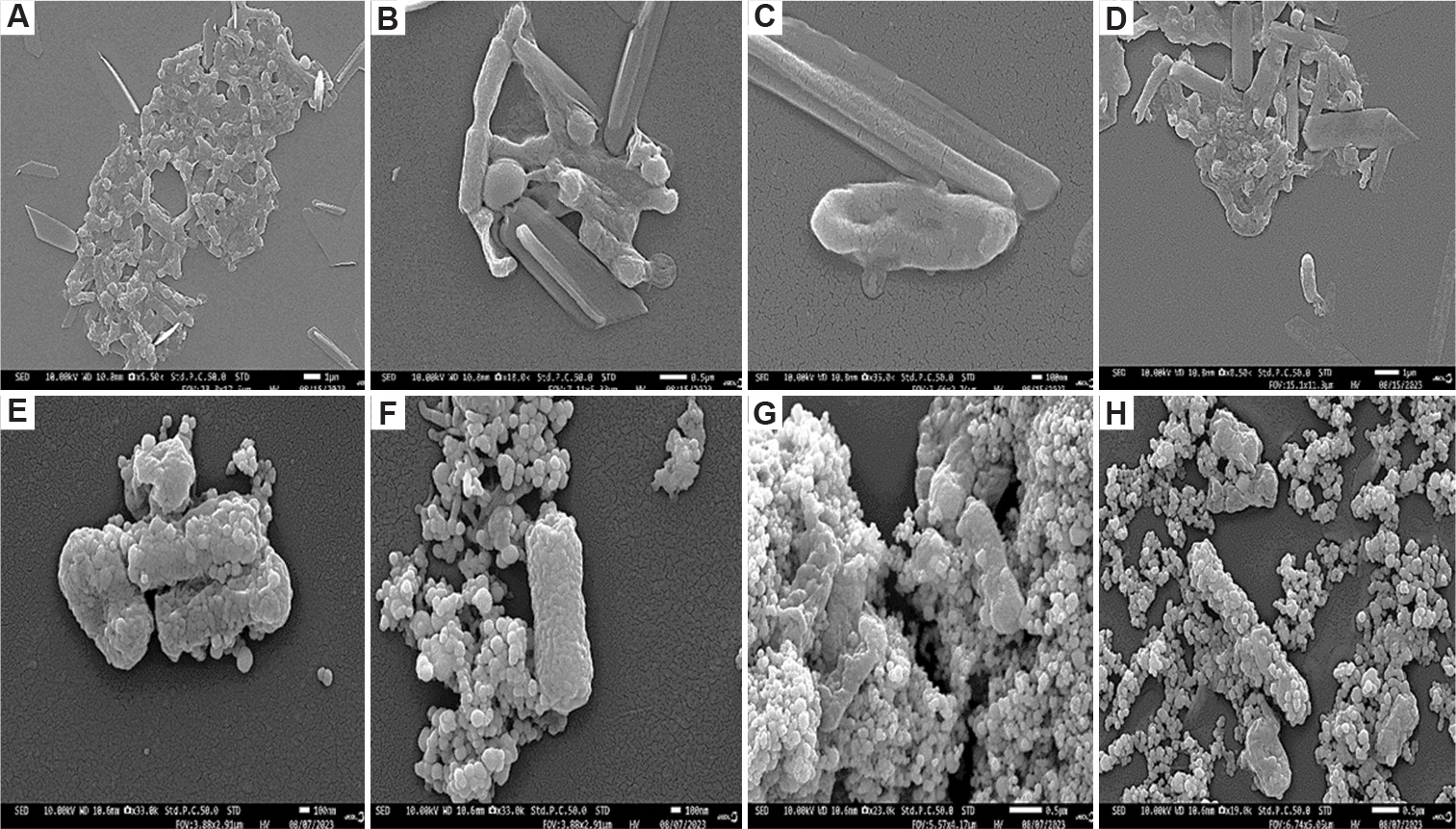
Scanning electron microscopy images of *Escherichia coli* cells show interactions with biosynthesized silver nanoparticles from carpenter bee wing extract after 24 h of exposure. (A-D) Control (untreated) cells; (E-H) cells treated with the silver nanoparticles. (A) Scale bar = 1 μm, magnification = ×5,500; (B) scale bar = 0.5 μm, magnification = ×10,000; (C) scale bar = 100 nm, magnification = ×33,000; (D) scale bar = 1 μm, magnification = ×8,500; (E and F) scale bar = 100 nm, magnification = ×33,000; (G) scale bar = 0.5 μm, magnification = ×23,000; (H) scale bar = 0.5 μm, magnification = ×19,000.

A decrease in optical density with increasing nanoparticle concentration suggests that silver nanoparticles inhibit the growth of *E. coli* and *K. pneumoniae* (Figure 3). To further investigate the bacterial response to silver nanoparticles, stress indicators, cell morphology, and nanoparticle–cell interactions were examined using SEM. The results show no aggregation in the control samples (i.e., in the absence of nanoparticles) (Figures 4A–D and 5A–D). However, aggregation is observed in *E. coli* and *K. pneumoniae* cells treated with silver nanoparticles (Figures 4E–H and 5E–H).

### 3.2. Genomic analysis

WGS analysis was conducted on control and treated *K. pneumoniae* and *E. coli* cells and compared against their respective reference genomes to identify potential genetic alterations and mutations resulting from exposure to biosynthesized silver nanoparticles after 24 h. The genomic variants identified in *K. pneumoniae* are presented in Tables 1 and 2. The treated cells display a total of four putative polymorphisms, three of which exceed a frequency of 0.5 (Table 1). These include mutations in the putrescine transport system adenosine triphosphate (ATP)-binding protein (*J2Y72_004072*), multidrug (MDR) efflux pump (*J2Y72_003942*), nitrate reductase beta subunit (*J2Y72_003241*), and ferric enterobactin receptor (*J2Y72_000218*).

The most significant polymorphisms identified in the control cells (Table 2) include: Staphyloferrin A export major facilitator superfamily transporter/D-ornithine citrate ligase (*sfaA/sfaD*), adenine phosphoribosyltransferase (*KQ76_RS08360*), teichoic acid D-alanine esterase (*fmtA*), DUF3169 family protein (*KQ76_RS01520*), alpha/beta hydrolase (*KQ76_RS13020*), DNA-binding heme response regulator (*hssR*), ribosome biogenesis guanosine triphosphate (GTP)-ase (*ylqF*), peptidoglycan teichoic acid D-alanyltransferase (*dltB*), M23 family metallopeptidase/haloacid dehalogenase-like hydrolase subfamily IIB (*KQ76_RS11280/KQ76_RS11285*), general stress protein (*KQ76_RS01815*), small stable RNA A-binding protein (*smpB*), phage major capsid protein (*KQ76_RS07375*), phosphoribosylformylglycinamidine synthase subunit (*purS*), transfer RNA uridine 5-carboxymethylaminomethyl (34) synthesis enzyme (*mnmG*), D-lactate dehydrogenase (*KQ76_RS12955*), and beta-glucoside operon antiterminator protein family transcriptional antiterminator (*KQ76_RS10985*).

WGS was conducted to identify polymorphisms in both control and treated cells following 24 h of exposure to biosynthesized silver nanoparticles. All detected polymorphisms, along with their mutation frequencies (f), are presented in Tables 2 and 3.

Single nucleotide polymorphisms identified in the control cells are presented in Table 4, along with descriptions of the de novo mutations. A total of 14 polymorphisms in the control cells are detected in the control cells, each showing a frequency increase ranging from 20% to 34%.

## 4. Discussion

Bacterial infections caused by *K. pneumoniae* and *E. coli* can result in serious, potentially life-threatening complications.^27,28^ Antibiotics remain powerful and lifesaving agents for treating infections such as urinary tract and bloodstream infections. However, *K. pneumoniae* and *E. coli* are increasingly developing resistance to antibiotics.^29^ As a result, nanoparticles have gained attention for their potential to combat bacterial resistance, owing to their unique physiochemical properties that enable multiple bactericidal mechanisms.^30^ The synthesis of nanoparticles represents a significant technological advancement, offering enhanced antimicrobial performance. Nevertheless, conventional synthesis methods may raise concerns related to toxicity and environmental impact. Therefore, the development of safe and sustainable nanoparticle production methods is essential.

Nanoparticles can be synthesized through various techniques, including chemical and biological (green) methods. Compared to chemical synthesis, green synthesis offers several advantages and is not associated with the limitations of chemical approaches.^31^ It is environmentally friendly, cost-effective, and offers potentially higher biocompatibility.^32^ In this study, silver nanoparticles were synthesized using CBWs, which act as a reducing agent for silver ions from silver nitrate. The study focuses on the synthesis, characterization, antibacterial evaluation, and mutation profiling in response to CBW-derived biosynthesized silver nanoparticles.

The mixing of CBW extract with silver nitrate results in a visible color change over time, indicating the reduction of silver ions and the excitation of the SPR peak associated with silver nanoparticles. Compounds such as aliphatic hydrocarbons in the CBW may facilitate the formation of silver nanoparticles within the size range of 20 – 40 nm.^25^ The UV-vis spectra of the synthesized nanoparticles show a peak at approximately 440 nm, which is characteristic of metal nanoparticles, consistent with findings from previous studies.^25–33^ Silver nanoparticles have been widely used as antimicrobial agents, demonstrating broad-spectrum efficacy against pathogens.^24,25,34^ In this study, the biosynthesized silver nanoparticles exhibit excellent antimicrobial activity by inhibiting the growth of *E. coli* and *K. pneumoniae*, likely through interactions with negatively charged components on the bacterial cell wall.^35^ The nanoparticles primarily adhere to the bacterial surface via electrostatic attraction and release positively charged silver ions, which disrupt cellular processes and damage DNA.^36^

Silver nanoparticles have the potential to induce mutations or polymorphisms, primarily through direct interaction with DNA and by generating oxidative stress.^37^ However, the mutagenic effects of silver nanoparticles and the associated resistance mechanisms in *E. coli* and *K. pneumoniae* remain largely unexplored. This study investigates whether biosynthesized silver nanoparticles from CBW can induce genetic mutations in *E. coli and K. pneumoniae*, potentially contributing to the development of resistance.

WGS, a technique that enables comprehensive identification of genomic mutations by sequencing an organism’s entire genome,^38^ was employed to analyze the interaction mechanisms between the biosynthesized silver nanoparticles and the bacterial cells. A key finding of this study is the detection of mutations in several genes of *K. pneumoniae*-treated cells that may reduce the antibacterial efficacy of silver nanoparticles. These mutations are associated with defense mechanisms, efflux systems, neutralization, ion transport, energy metabolism, and siderophore production.

Notably, mutations are identified in the genes encoding the putrescine transport system ATP-binding protein (*J2Y72_004072*), MDR pump (*J2Y72_003942*), nitrate reductase beta subunit (*J2Y72_003241*), and ferric enterobactin receptor (*J2Y72_000218*). Among these, the mutation in the ATP-binding cassette (ABC) transporter gene (*J2Y72_004072*) is particularly significant, as it exhibits the highest mutation frequency (100%).

In WGS, mutation frequency refers to the proportion of a specific genetic variation observed within the studied population. ABC transporters are responsible for importing nutrients and exporting toxic substances in bacterial cells.^39^ Mutations in ABC transporters can contribute to antimicrobial resistance, thereby reducing the efficacy of silver nanoparticles. This study suggests that exposure to silver nanoparticles may promote the emergence of such mutations in ABC transporter genes. Mutations in ABC transporters can significantly affect bacterial physiology by disrupting nutrient uptake or causing uncontrolled efflux of vital intracellular components. These disruptions can impair growth, alter virulence, and modulate antibiotic susceptibility.^39^ Ultimately, the inability to maintain intracellular homeostasis may compromise cellular processes, leading to reduced bacterial viability or cell death.

Other genomic variants identified in *K. pneumoniae* exposed to biosynthesized silver nanoparticles are associated with transport and resistance mechanisms, including mutations in genes encoding for MDR pump (*J2Y72_003942*), nitrate reductase beta subunit (*J2Y72_003241*), and ferric enterobactin receptor (*J2Y72_000218*). MDR pumps are membrane-associated transporter proteins that expel toxic compounds from bacterial cells, enhancing survival and contributing to antibiotic resistance.^40^ These pumps also protect bacteria from antimicrobial agents and harmful substances, including heavy metals and organic solvents.^41^ The nitrate reductase beta subunit forms part of an enzyme complex involved in electron transfer and energy production,^40^ whereas the ferric enterobactin receptor is an outer membrane protein responsible for transporting iron into the periplasm.^42–45^ Mutations affecting iron transport systems can lead to antimicrobial resistance by impairing iron uptake. This is significant because many antibiotics rely on iron transport pathways to enter bacterial cells.^46^ Consequently, limiting iron acquisition can enhance bacterial resistance to antimicrobial agents.

The nutrient broth medium provides a rich source of readily available nutrients – such as carbohydrates, protein, vitamins, and minerals – that enable *K. pneumoniae* to efficiently access nutrients necessary for rapid growth and proliferation.^25^ In the control group, mutations are detected in *K. pneumoniae* cells grown in this nutrient-rich medium. These mutations appear to confer advantageous traits that enhance nutrient utilization, allowing the control cells to outcompete the treated cells and display increased growth.

Several notable mutations are observed in the control cells, particularly in genes related to iron metabolism, biosynthesis, metabolism, cell growth, detoxification, cell wall integrity, structural stability, defense, and stress responses. These mutations likely provide a competitive advantage in the nutrient-rich media. This also raises concerns about stress-induced mutagenesis, as the observed mutations in the control group may reflect an elevated rate of adaptive mutation – potentially contributing to future resistance development in *K. pneumoniae.* Nevertheless, the study demonstrates that biosynthesized silver nanoparticles effectively inhibit bacterial growth, suggesting a cytotoxic effect of silver nanoparticles that interferes with essential cellular processes and disrupts normal cell function.

Mutations can arise spontaneously without exposure to external stressors and are a key driver of bacterial evolution. In untreated bacterial cells, mutations may result from natural genetic alterations during DNA replication. While many of these changes are neutral, some may confer advantageous traits – such as increased antibiotic resistance – that enhance bacterial survival in challenging environments, including exposure to antimicrobials.^12^

These findings highlight a critical concern: the presence of resistance genes may render nanoparticles ineffective. Such genes can reduce nanoparticle efficacy through several mechanisms, including actively expelling nanoparticles, modifying the cell membrane to prevent nanoparticle entry, and chemically altering nanoparticles to reduce their toxicity.

Genomic analysis reveals several mutations in the genes of control *K. pneumoniae* cells, including *sfaA/sfaD*, *KQ76_RS08360*, *fmtA*, *KQ76_RS01520*, *KQ76_RS13020*, *hssR*, *ylqF*, *dltB*, *KQ76_RS11280/KQ76_RS11285*, *KQ76_RS01815*, *smpB*, *KQ76_RS07375*, *purS*, *mnmG*, *KQ76_RS12955*, and *KQ76_RS10985*.

Describing the function of these genes is crucial for understanding how *K. pneumoniae* adapts to its environment, particularly in relation to antibiotic resistance, pathogenicity, and microbial evolution. Mutations in these genes can significantly alter bacterial traits by affecting key cellular processes such as metabolism, virulence factor expression, and drug susceptibility.

The following are the functions of the mutated genes identified in *K. pneumoniae* control cells:

- *sfaA/sfaD* is involved in the transport of iron from the environment into the cell, supporting essential cellular processes.^47^
- *KQ76_RS08360* enables the recycling of adenine, a critical building block of DNA.^48^
- *fmtA* is involved in cell division and bacterial cell wall synthesis.^49^
- *KQ76_RS13020* belongs to a large enzyme superfamily with diverse catalytic functions,^50^ including roles in cell growth, metabolism, and detoxification.
- *hssR* regulates gene expression related to iron metabolism and other cellular activities.^51^
- *ylqF* assists in the assembly and regulation of ribosomes. GTPases also regulate cellular functions.^52^
- *dltB* maintains cell wall integrity and regulates cation balance, contributing to resistance against cationic antimicrobial peptides.^53^
- *KQ76_RS11280/KQ76_RS11285* facilitates bacterial competition for resources or consumption of other bacteria.^54^
- *KQ76_RS01815* promotes bacterial survival under environmental stresses and induces virulence factor expression.^55^
- *smpB* is involved in tagging and degrading proteins produced from defective mRNAs and plays a role in nutrient acquisition.^56^
- *KQ76_RS07375* triggers bacterial defense mechanisms.^57^
- *purS* is involved in the purine biosynthetic pathway.^58^
- *mnmG* is crucial for accurate codon-anticodon pairing during protein translation.^59^
- *KQ76_RS12955* is a key enzyme in glycolysis.^60^

Mutations identified in *E. coli*-treated cells involve genes associated with transport, cell division, biosynthetic adaptation, and invasion:

- L-lysine exporter LysO/aquaporin Z (*lysO/aqpZ*) mediates the export of L-lysine and confers resistance to the toxic antimetabolite L-thialysine.^61^
- YtfJ family protein (*D1792_RS11465*) is involved in cell division and cell wall hydrolysis.^62^
- Succinate-coenzyme A ligase subunit alpha (*sucD*) plays a role in ATP synthesis.^63^
- Histidinol dehydrogenase (*hisD*) is essential for bacterial survival.^64^
- Helix-turn-helix transcriptional regulator (*D1792_RS02575*) modulates gene expression by activating or repressing transcription.^65^
- Proton symporters (*uacT*) transport substrates and protons across the cell membrane, aiding bacterial adaptation to environmental changes.^66^
- Host specificity proteins (*D1792_RS03370*) contribute to bacterial infectivity and assist in evading the host immune response.^67^
- Major facilitator superfamily transporters (*ygcS*) help bacteria withstand toxic metabolites, heavy metals, and environmental stressors.^68^

The control cells exhibit a distinct mutation pattern compared to the treated cells. *E. coli* control cells carry mutations in genes such as intermembrane transport protein (*pqiB*), glucans biosynthesis protein (*mdoG*), invasion regulator (*sirB2*), phosphotransferase system fructose transporter subunit (*IIC*), *hisD*, phosphoglycerate dehydrogenase/sugar isomerase domain-containing protein (*D1792_RS10070/D1792_RS10075*), bifunctional chitinase/lysozyme (*chiA*), LysR family transcriptional regulator (*rcdB*), and initiator associating protein (*diaA*). These mutations influence *E. coli* growth by altering functions related to nutrient acquisition, stress response, immune evasion, or antibiotic resistance, as supported by their known roles.

*pqiB* is essential for bacterial survival, pathogenesis, and antimicrobial resistance.^69^ *mdoG* modulates virulence, biofilm structure, and immune evasion.^70^ *sirB2* supports bacterial survival and adaptation.^71^ *IIC* facilitates sugar transport across the membrane.^72^ *hisD* catalyzes the final two steps in histidine biosynthesis and is vital for survival during infection.^64^ *D1792_RS10070/D1792_RS10075* produces serine, a key amino acid for protein production.^73^ *chiA* acts as a virulence factor by allowing *E. coli* to invade chitinous hosts – such as insects or fungi – through cell wall degradation.^74^ *rcdB* contributes to metabolism, stress response, and virulence.^75^ *diaA* acts as the primary “initiator” protein.^76^

Mutations in *lysO/aqpZ* and *D1792_RS11465* are shared between control and treated groups. *lysO/aqpZ* mediates L-lysine export and confers resistance to the toxic antimetabolite L-thialysine,^61^ while *D1792_RS11465* is involved in cell division and cell wall hydrolysis.^72^ Collectively, these mutations enhance *E. coli*’s ability to survive under challenging conditions by improving resource utilization, antibiotic resistance, and immune evasion.

The findings indicate that *K. pneumoniae* is more sensitive to silver nanoparticles than *E. coli* (Figure 2), possibly due to structural differences in their cell walls. *E. coli* possesses a relatively thicker peptidoglycan layer, which can hinder nanoparticle penetration, whereas the thinner cell wall of *K. pneumoniae* allows easier entry and interaction with the cell membrane.^77^ Furthermore, variations in lipopolysaccharides (LPS) between *E. coli* and *K. pneumoniae* may influence nanoparticle aggregation and uptake. LPS, present on the surface of Gram-negative bacteria, are known to attract and bind to nanoparticles.^78^ The LPS of *K. pneumoniae* typically has a more complex structure with additional sugar modifications compared to those of *E. coli*, potentially enhancing their interaction with silver nanoparticles and contributing to increased sensitivity.^79^ The highly charged and hydrophilic nature of *K. pneumoniae* LPS promotes strong binding to nanoparticle surfaces, which may disrupt the bacterial outer membrane and compromise cell viability.^80^

## 5. Conclusion

Nanoparticles hold great promise as antimicrobial agents due to their potent antibacterial activity, particularly when synthesized using metals such as silver. CBW-derived silver nanoparticles are highly effective against *E. coli* and *K. pneumoniae* by aggregating on the bacterial cell surface. These biosynthesized nanoparticles present a suitable alternative to conventional antibiotics for addressing antibiotic resistance in *E. coli* and *K. pneumoniae* and are strong candidates for medical applications where antimicrobial activity is essential. Future studies should investigate the potential toxicity of biosynthesized silver nanoparticles on human cells to ensure their safe application for both human health and the environment.

## Figures and Tables

**Table 1.** Genomic analysis of *Klebsiella pneumoniae* cells after 24 h of exposure to biosynthesized silver nanoparticles from carpenter bee wing extract

Position	Frequency (%)	Annotation	Gene	Product

4,398,960	100.0	S189A* (TCG→GCG)	*J2Y72_004072*	Putrescine transport system ATP-binding protein
4,264,009	62.3	I879M* (ATT→ATG)	*J2Y72_003942*	Multidrug efflux pump
3,539,179	61.1	K215K* (AAG→AAA)	*J2Y72_003241*	Nitrate reductase beta subunit
227,265	55.6	Coding (303/2229 nt)	*J2Y72_000218*	Ferric enterobactin receptor

Notes: Asterisk (*) indicates that the annotation provides functional context to the corresponding gene sequence, facilitating interpretation and analysis; Underlined letters denote specific nucleotide or amino acid mutations identified within the sequence.

Abbreviation: ATP: Adenosine triphosphate.

**Table 2.** Genomic analysis of *Klebsiella pneumoniae* cells in the control group

Position	Frequency (%)	Annotation	Gene	Product
2,228,365	69.2	Intergenic (−35/−67)	*sfaA/sfaD*	Staphyloferrin A export MFS transporter/ D-ornithine citrate ligase
1,700,478	65.6	G59S* (GGC→AGC)	*KQ76_RS08360*	Adenine phosphoribosyltransferase
1,017,325	65.3	Q304* (CAA→TAA)	*fmtA*	Teichoic acid D-alanine esterase
342,330	62.0	I180I* (ATT→ATC)	*KQ76_RS01520*	DUF3169 family protein
2,574,726	32.2	G69A* (GGC→GCC)	*KQ76_RS13020*	Alpha/beta hydrolase
2,389,192	32.0	R188P* (CGA→CCA)	*hssR*	DNA-binding heme response regulator
1,213,003	31.3	E184D* (GAG→GAC)	*ylqF*	Ribosome biogenesis GTPase
860,322	31.3	E344K* (GAA→AAA)	*dltB*	Peptidoglycan teichoic acid D-alanyltransferase
2,252,747	29.9	Intergenic (−54/+157)	*KQ76_RS11280/KQ76_RS11285*	M23 family metallopeptidase/haloacid dehalogenase-like hydrolase subfamily IIB
391,361	29.1	S36I *(AGT→ATT)	*KQ76_RS01815*	General stress protein
810,694	29.0	M1K* (ATG→AAG)	*smpB*	SsrA-binding protein
1,536,460	27.5	Y112* (TAT→TAA)	*KQ76_RS07375*	Phage major capsid protein
1,027,271	27.0	A87P* (GCA→CCA)	*purS*	Phosphoribosylformylglycinamidine synthase subunit
2,776,116	26.7	H117Q* (CAT→CAA)	*mnmG*	tRNA uridine 5-carboxymethylaminomethyl (34) synthesis enzyme
2,574,727	26.6	G69R* (GGC→CGC)	*KQ76_RS13020*	Alpha/beta hydrolase
2,564,194	26.6	A17P* (GCA→CCA)	*KQ76_RS12955*	D-lactate dehydrogenase
2,190,680	25.7	T500S* (ACG→TCG)	*KQ76_RS10985*	BglG family transcriptional antiterminator

Notes: Asterisk (*) indicates that the annotation provides functional context to the corresponding gene sequence, facilitating interpretation and analysis; Underlined letters denote specific nucleotide or amino acid mutations identified within the sequence.

Abbreviations: BglG: Beta-glucoside operon antiterminator protein; GTP: Guanosine triphosphate; MFS: Major facilitator superfamily; SsrA: Small stable RNA A; tRNA: Transfer RNA.

**Table 3.** Genomic analysis of *Escherichia coli* after 24 h of exposure to biosynthesized silver nanoparticles from carpenter bee wing extract

Position	Frequency (%)	Annotation	Gene	Product
4,935,197	43.3	Intergenic (−347/+147)	*lysO/aqpZ*	L-lysine exporter LysO/aquaporin Z
2,376,506	42.9	E119* (GAA→TAA)	*D1792_RS11465*	YtfJ family protein
4,790,571	28.2	A218P* (GCA→CCA)	*sucD*	Succinate-CoA ligase subunit alpha
1,244,541	25.4	V392L* (GTA→CTA)	*hisD*	Histidinol dehydrogenase
461,346	25.0	R159P* (CGG→CCG) ‡	*D1792_RS02575*	Helix-turn-helix transcriptional regulator
2,155,046	22.9	T314R* (ACG→AGG)	*uacT*	Urate/proton symporter UacT
641,105	21.1	E1049D* (GAG→GAC)	*D1792_RS03370*	Host specificity protein J
1,997,598	20.7	V23L* (GTA→CTA)	*ygcS*	MFS transporter

Notes: Asterisk (*) indicates that the annotation provides functional context to the corresponding gene sequence, facilitating interpretation and analysis; Double dagger (‡) indicates a variant that is flagged as potentially problematic or requires further investigation; Underlined letters denote specific nucleotide or amino acid mutations identified within the sequence.

Abbreviations: CoA: Coenzyme A; MFS: major facilitator superfamily; UacT: Uric acid transporter.

**Table 4.** Genomic analysis of *Escherichia coli* cells in the control group

Position	Frequency (%)	Annotation	Gene	Product
5,022,977	35.4	L28V* (CTC→GTC)	*pqiB*	Intermembrane transport protein PqiB
82,367	29.6	A100P* (GCC→CCC)	*mdoG*	Glucan biosynthesis protein G
5,022,975	29.2	A27G* (GCG→GGG)	*pqiB*	Intermembrane transport protein PqiB
4,935,212	28.6	Intergenic (−362/+132)	*lysO/aqpZ*	L-lysine exporter LysO/aquaporin Z
325,706	27.4	S9R* (AGC→AGG)	*sirB2*	Invasion regulator SirB2
1,664,261	27.1	T163T* (ACC→ACG)	*fryC*	PTS fructose transporter subunit IIC
1,244,541	26.2	V392L* (GTA→CTA)	*hisD*	Histidinol dehydrogenase
1,078,228	25.0	A53P* (GCC→CCC)	*D1792_RS05680*	DUF4756 family protein
2,077,731	24.0	Intergenic (−7/+65)	*D1792_RS10070/D1792_RS10075*	Phosphoglycerate dehydrogenase/SIS domain-containing protein
2,376,513	23.8	G116G* (GGC→GGA)	*D1792_RS11465*	YtfJ family protein
2,725,980	22.5	G94G* (GGC→GGG)	*chiA*	Bifunctional chitinase/lysozyme
2,936,459	21.8	L94* (TTA→TGA) ‡	*rcdB*	LysR family transcriptional regulator
2,555,115	21.5	V98L* (GTG→CTG)	*diaA*	DnaA initiator-associating protein DiaA
2,077,733	20.0	Intergenic (−9/+63)	*D1792_RS10070/D1792_RS10075*	Phosphoglycerate dehydrogenase/SIS domain-containing protein

Note: Asterisk (*) indicates that the annotation provides functional context to the corresponding gene sequence, facilitating interpretation and analysis; Double dagger (‡) indicates a variant that is flagged as potentially problematic or requires further investigation; Underlined letters denote specific nucleotide or amino acid mutations identified within the sequence.

Abbreviations: PTS: Phosphotransferase system; PqiB: Paraquat-inducible protein B; SirB2: Signal regulatory protein beta 2; SIS: Sugar isomerase.

## Data Availability

The data that support the findings of this study are available upon request from the corresponding author.
